# Effect of thyroid dysfunction on N-terminal pro-B-type natriuretic peptide levels: A systematic review and meta-analysis

**DOI:** 10.3389/fendo.2023.1083171

**Published:** 2023-01-26

**Authors:** Hongling Zhang, Xiaotao Li, Nawen Zhang, Limin Tian

**Affiliations:** ^1^ The First Clinical Medical College of Gansu University of Chinese Medicine (Gansu Provincial Hospital), Lanzhou, China; ^2^ Department of Endocrinology, Gansu Provincial Hospital, Lanzhou, China; ^3^ The First School of Clinical Medicine, Lanzhou University, Lanzhou, China; ^4^ Clinical Research Center for Metabolic Diseases, Lanzhou, Gansu, China

**Keywords:** NT-ProBNP, thyroid dysfunction, heart failure, systematic review, meta-analysis

## Abstract

**Purpose:**

Thyroid hormones (THs) significantly affect the cardiovascular system. N-terminal pro-B-type natriuretic peptide (NT-proBNP) is a useful biomarker for diagnosing, evaluating, and predicting outcomes in heart failure (HF). This comprehensive review and meta-analysis aimed to investigate the effects of thyroid dysfunction (hypothyroidism and hyperthyroidism) on NT-proBNP levels.

**Methods:**

Two investigators independently searched PubMed, Embase, Cochrane Library, and Web of Science databases for studies published from inception to July 31, 2022, without any restrictions on language.

**Results:**

21 studies were included. In participants without HF, NT-proBNP levels may be elevated in those with overt hyperthyroidism (standardized mean difference [SMD] 2.38, 95% confidence interval [CI]:1.0-3.76). Notably, among patients with preexisting HF, significantly higher NT-proBNP levels were found in patients with overt hyperthyroidism, overt hypothyroidism, or subclinical hypothyroidism than in euthyroid subjects (SMD [95%CI] = 0.31[0.01, 0.62], 0.32[0.08, 0.56], and 0.33[0.21, 0.46], respectively). Seven trials compared NT-proBNP levels in patients with thyroid dysfunction before and after therapy, and significant drops in NT-proBNP levels were observed in patients with hyperthyroidism (SMD [95%CI] = -1.53[-2.50, -0.55]) upon achieving a euthyroid state. In contrast, increased NT-proBNP levels were observed in hypothyroid patients after treatment (SMD [95%CI] = 1.07[0.28, 1.85]).

**Conclusion:**

Thyroid dysfunction can significantly affect NT-proBNP levels, which may change upon achieving a euthyroid state. Notably, the effect of thyroid dysfunction on cardiac function may depend on the underlying cardiac status. Thus, timely recognition and effective treatment of cardiac symptoms in patients with thyroid dysfunction are mandatory because the prognosis of HF may be improved with appropriate treatment of thyroid dysfunction.

**Systematic review registration:**

https://www.crd.york.ac.uk/prospero, identifier CRD42022353700.

## Introduction

Thyroid dysfunction (TD) may occur because of hypothyroidism or hyperthyroidism. Serum thyroid stimulating hormone (TSH) levels are increased with normal (subclinical hypothyroidism, SHypo) or low serum free thyroxin (FT4) (overt hypothyroidism, OHypo) levels, whereas serum TSH levels are low with normal (subclinical hyperthyroidism, SHyper) or high FT4 (overt hyperthyroidism, OHyper) levels. As thyroid hormones play an important role in regulating cardiac activities and affecting cardiovascular hemodynamics, thyroid conditions can cause metabolic and hemodynamic changes that may result in heart failure (HF) (1).

B-type natriuretic peptide (BNP) is a cardiac neurohormone generated by ventricles in response to volume expansion or pressure overload. BNP and N-terminal prohormone of brain natriuretic peptide (NT-proBNP) are two types of natriuretic peptides cleaved from Pro brain natriuretic peptide (proBNP). Compared to BNP, NT-proBNP is more stable and has a longer biological half-life. Thus, NT-proBNP is a better indicator for diagnosing or ruling out HF (2). NT-proBNP is also a good marker for assessing the severity and prognosis of this condition (3). Some studies have revealed that TD may affect serum NT-proBNP levels, but no consensus has been reached. Several studies (4–6) demonstrated that OHypo patients had significantly elevated NT-proBNP levels compared to euthyroid patients, but some studies found no correlation between them (7–9). Pakula et al. (9) observed a significant increase in NT-proBNP levels in SHyper patients, but Christ et al. (7) reported no such increase in SHyper patients compared to subjects in the control group. Furthermore, Hadzovic et al. (10) found that treating hypothyroidism resulted in a significant elevation of NT-proBNP levels, which appears inconsistent with the findings of Schultz et al. (11). Therefore, this meta-analysis aimed to investigate the effect of TD on NT-proBNP levels.

## Methods

This meta-analysis followed the Preferred Reporting Items for Systematic Reviews and Meta-Analyses (PRISMA) statement (12), and this systematic review was registered in the International Prospective Register of Systematic Reviews (PROSPERO), CRD42022353700.

### Data sources and search strategy

Two investigators independently searched for studies in databases including PubMed, Embase, Cochrane Library, and Web of Science from inception to July 31, 2022, without language restrictions. The search strategies (**Table S1** of the **Supplementary Materials**) were (hypothyroidism* OR hyperthyroidism* OR thyroid dysfunction) AND (BNP OR NT-proBNP).

### Literature screening

Inclusion criteria: 1) studies comparing NT-proBNP levels in TD subjects and euthyroid subjects; clinical trials that compared NT-proBNP levels at pre-to-post treatment in TD; 2) studies reporting TD according to thyroid function test results; and 3) NT-proBNP levels in patients were reported as mean ± standard deviation (or calculable).

The exclusion criteria were as follows: 1) participants from a specific population (e.g., children or pregnant women); 2) studies investigating the effect of TD on BNP levels instead of NT-proBNP levels; and 3) reviews, conference abstracts, case reports, and studies with unavailable full texts.

Two researchers conducted the literature screening independently, and disagreements were resolved through discussion with a third researcher.

### Data extraction and quality assessment

Two researchers independently performed data extraction, and any disagreements were settled through discussion with a third researcher. Extracted information was as follows: first author, publication year, country, sample size, sex, age, type of thyroid dysfunction, LVEF%, NT-proBNP detection method, TSH, FT3, FT4, and NT-proBNP levels in subjects with euthyroid, TD, and pre-to-post treatment in TD.

Given the types of included studies (case-control and cohort studies), the Newcastle-Ottawa Scale (NOS) was used to assess the quality of the included studies (13). The score ranges from 0 to 9; 7–9 represent high-quality scores, 4–6 represent medium scores and 1–3 represent low scores. Self-controlled trials that discussed NT-proBNP at pre-to-post treatment in TD were assessed using the JBI critical appraisal tool for quasi-experimental studies (14).

### Collection and interpretation of data

We extracted the data on NT-proBNP levels in TD and euthyroid subjects, as well as at pre-to-post treatment in TD. Continuous variables were reported as standardized mean differences (SMDs) with a 95% confidence interval (CI). The chi-squared-based Q test and the *I^2^* test were performed to evaluate the heterogeneity across included studies, and *I^2^* ≤50% and *I^2^* >50% indicated low and high levels of heterogeneity, respectively. If there was a low level of heterogeneity, a fixed-effects model was used to pool data. Otherwise, a random effects model was used. Since some studies compared NT-proBNP levels in TD patients with HF and euthyroid patients with HF, subgroup analyses were performed according to whether the patients had HF. A sensitivity analysis was performed by sequentially removing each study. Publication bias was evaluated using a funnel plot, and if the included studies had an outcome of more than 10. All statistical tests were two-sided, and the significance level was set at p < 0.05. Review Manager software (Version 5.4.1, The Cochrane Collaboration, 2020) was used to conduct the meta-analysis.

## Results

### Study identification and selection

**Figure 1:**
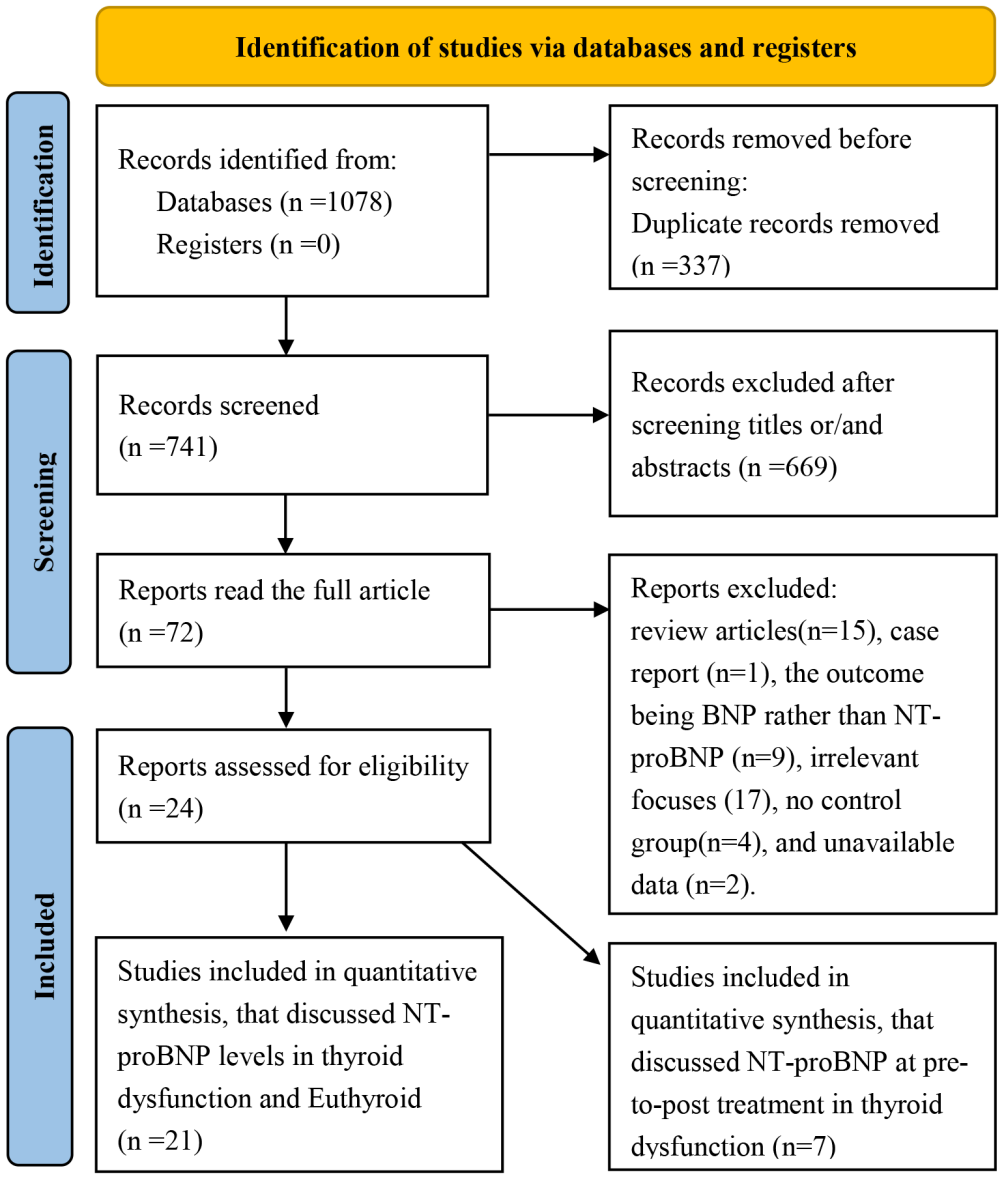
PRISMA flow diagram of this systematic review and meta-analysis.

The study selection process is shown in **Figure 1**. After duplicates were removed, titles and abstracts were screened, and 72 studies were obtained. After a comprehensive review of full texts, 48 articles were excluded for the following reasons: review articles (n=15), case reports (n=1), the outcome being BNP rather than NT-proBNP (n=9), irrelevant focuses (n=17), no control group (n=4), and unavailable data (n=2). Finally, 24 papers were included in this meta-analysis.

### Characteristics and quality of included studies

Among the 24 included studies, 21 (4–10, 15–28) compared NT-proBNP levels in TD and euthyroid subjects. The characteristics of the subjects in the TD and euthyroid groups are summarized in **Table 1**. The NOS results are presented in **Table 1** and **Table S2** (**Supplementary Materials**). The results showed that these studies were of medium-to-high quality. **Table 2** shows the characteristics of self-controlled trials (7, 9–11, 19, 29, 30) that discussed NT-proBNP levels at pre-to-post treatment in TD patients. The prior cardiovascular disease in patients with HF in ten studies (6, 20–28) is summarized in **Table S3**. The JBI critical appraisal tool was employed for quasi-experimental studies to assess the quality of the self-controlled trials, and we found the following reasons for lower study quality: 1) all studies had no control group, and 2) results were not measured multiple times. In general, the included self-controlled trials were of high quality (**Table S4** of the **Supplementary Materials**).

**Table 1:** General characteristics of the included studies discussing NT-proBNP levels in TD and euthyroid.

Author, year	Region	Cohort	Sample size	Sex(F/M)	Age (range or mean ± sd)	TSH (mIU/L)	FT3	FT4	LVEF(%)	NT-proBNP method	Score
Christ 2005 (7)	Switzerland	SHypo	63	63/0	58 ± 10	11 ± 6	1.9 ± 0.4 *	12 ± 2 #	N/A	ECLIA	8
		OHypo	35	35/0	55 ± 12	45 ± 24	1.2 ± 0.6 *	5 ± 2 #			
		SHyper	14	14/0	51 ± 11	0.02 ± 0.01	2.2 ± 0.4 *	20 ± 2 #			
		OHyper	10	10/0	55 ± 11	<0.01	3.6 ± 2.0 *	37 ± 9 #			
		Euthyroid control	40	40/0	51 ± 12	1.7 ± 0.6	1.7 ± 0.3 *	15 ± 3 #			
Arikan 2007 (8)	Turkey	OHypo	25	22/3	35.4 ± 13.9	29.67 ± 5.74	4.16 ± 1.39 #	11.84 ± 5.27 #	61.30 ± 2.0	ECLIA	7
		OHyper	36	27/9	42.9 ± 16.7	0.05 ± 0.06	31.57 ± 2.31#	51.22 ± 13.12 #	60.29 ± 6.26		
		Euthyroid control	34	18/16	41.4 ± 13.8	1.54 ± 0.73	5.23 ± 0.46 #	15.71 ± 66.8 #	61.76 ± 2.46		
Ozmen 2007 (15)	Turkey	OHypo	24	17/7	49.33 ± 7.31	43.46 ± 9.66	2.78 ± 0.66 #	7.76 ± 1.46 #	N/A	ECLIA	6
		OHyper	21	16/5	50.05 ± 7.00	<0.01	13.38 ± 4.34 #	47.51 ± 7.07 #			
		Euthyroid control	20	14/6	47.04 ± 7.72	1.32 ± 0.39	4.61 ± 0.49 #	14.52 ± 2.79 #			
Gu 2011 (16)	China	OHyper	239	182/57	37.3 ± 0.83	0.564 ± 0.155	14.73 ± 0.89 #	26.95 ± 1.10 #	N/A	ELISA	8
		Euthyroid control	81	63/18	35.3 ± 0.8	0.741 ± 0.083	4.21 ± 0.05 #	13.64 ± 0.14 #			
Hadzovic 2011 (10)	Bosnia and Herzegovina	OHypo	35	35/0	49.1 ± 4.3	13.29 ± 1.09	3.34 ± 0.29 *	8.28 ± 0.69 *	N/A	ECLIA	6
		OHyper	34	34/0	41.6 ± 10.0	0.03 ± 0.08	12.75 ± 1.66 *	28.16 ± 2.78 *			
		Euthyroid control	35	35/0	43.7 ± 8.8	1.75 ± 0.23	5.2 ± 0.21 *	15.3 ± 0.37 *			
Pakuła 2011 (9)	Poland	SHypo	14	N/A	54.71 ± 18.71	7.012 ± 2.287	2.29 ± 0.75 !	1.306 ± 0.724 !	N/A	immunoenzymatic method	5
		OHypo	24		57.46 ± 15.24	29.901 ± 21.902	1.89 ± 0.89 !	0.625 ± 0.381 !			
		SHyper	16		59.37 ± 16.03	0.079 ± 0.153	3.11 ± 0.94 !	1.335 ± 0.518 !			
		OHyper	47		52.79 ± 13.74	0.047 ± 0.122	11.49 ± 7.01 !	4.434 ± 2.107 !			
		Euthyroid control	30		59.96 ± 12.82	1.808 ± 1.036	2.76 ± 0.72 !	1.297 ± 0.28 !			
Schultz 2011 (17)	Denmark	SHypo	31	26/5	69.5 ± 10	5.84 (4.49–7.35)	N/A	N/A	N/A	ECLIA	6
		Shyper	25	20/5	74 ± 10	0.26 (0.12–0.34)					
		Euthyroid control	549	306/243	67.5 ± 10.5	1.36 (0.93–1.95)					
Ulusoy 2013 (4)	Turkey	OHypo	28	24/4	46.15 ± 11.91	>10	<1.71 !	0.7 !	N/A	ECLIA	6
		OHyper	25	16/9	34.90 ± 11.49	<0.35	>3.71 !	>1.48 !			
		Euthyroid control	40	23/17	39.03 ± 12.37	N/A	N/A	N/A			
Jiang 2016 (18)	China	OHypo	229	239/233	79.8 ± 10.5	N/A	N/A	N/A	N/A	ECLIA	6
		OHyper	9		69 ± 8.1						
		Euthyroid control	234		74.3 ± 10.6						
Muthukumar 2016 (19)	India	OHyper	41	N/A	39.4 ± 8.6	N/A	10.2 ± 3.8 !	2.2 ± 0.9 &	59.34 ± 6.48	N/A	7
		Euthyroid control	20		40.6 ± 9.1	N/A	2.9 ± 0.7 !	1.2 ± 0.2 &	63 ± 2.17		
Cozma 2017 (5)	Romania	OHypo	34	34/0	47 ± 5.1	6.8 ± 2.4	N/A	0.56 ± 0.09 !	59 ± 5.8	Immunoenzymatic method	8
		OHyper	30	30/0	46.3 ± 5.8	0.14 ± 0.1		1.91 ± 0.33 !	65.7 ± 8.2		
		Euthyroid control	30	30/0	45.7 ± 4.9	2.65 ± 0.8		0.96 ± 0.17 !	67.4 ± 6.8		
Iacoviello 2008 (25)	Italy	SHypo with HF	34	9/25	69 ± 10	11.0 ± 7.5	2.8 ± 0.6 !	1.2 ± 0.3 !	31 ± 11	immunoassay	6
		Euthyroid with HF	304	70/234	64 ± 13	1.9 ± 1.1	3.1 ± 0.4 !	1.3 ± 0.2 !	32 ± 9		
Li 2014 (20)	China	SHypo with HF	79	25/54	53.2 ± 13.7	8.52 (6.42-13.92)	2.67 ± 0.53 !	1.22 (1.03-1.43) !	31.5 ± 8.1	N/A	6
		SHyper with HF	68	23/45	56.9 ± 14.5	0.2 (0.055-0.29)	3.13 ± 2.07 !	1.355(1.1925-1.6175) !	29.7 ± 7.5		
		Euthyroid with HF	816	205/611	51.6 ± 14.5	1.71 (1.07-2.69)	2.80 ± 0.51 !	1.32 (1.17-1.51) !	32.4 ± 8.5		
Perez 2014 (21)	Scotland; Norway; United Kingdom	OHypo with HF	237	55/182	73.0 ± 6.8	9.65 ± 15.71	N/A	N/A	30.5 ± 6.7	N/A	7
		OHyper with HF	175	53/122	72.9 ± 6.2	0.14 ± 0.10			31.3 ± 6.5		
		Euthyroid with HF	4338	953/3385	72.6 ± 7.1	1.82 ± 1.01			30.9 ± 6.5		
Berezin 2015 (22)	Ukraine	SHypo with HF	53	25/28	58.81 ± 6.50	18.62 (11.92-25.4)	5.98 (4.63-7.87) #	13.8 (10.9-18.0) *	42.31 ± 3.54	ECLIA	7
		Euthyroid with HF	335	156/179	57.26 ± 6.90	3.86 (2.36-4.57)	5.85 (4.06-7.44) #	12.9 (9.6-15.9) *	43.60 ± 4.55		
Wang 2015 (23)	China	SHypo with HF	41	12/29	48 ± 14	8.59 ± 4.91	2.72 ± 0.53 !	1.34 ± 0.28 !	28 ± 8	N/A	6
		OHypo with HF	12	6/6	49 ± 15	36.75 ± 24.24	1.48 ± 0.25 !	0.71 ± 0.16 !	28 ± 7		
		SHyper with HF	35	7/28	52 ± 10	0.36 ± 0.16	2.72 ± 0.48 !	1.39 ± 0.29 !	32 ± 7		
		Euthyroid with HF	353	131/222	51 ± 14	2.00 ± 1.06	2.80 ± 0.47 !	1.35 ± 0.26 !	33 ± 10		
Hazem 2018 (24)	Egypt	OHyper with HF	30	17/13	58.74 ± 3.22	0.02 ± 0.09	7.2 ± 1.4 #	41.8 ± 12.6 #	40 (36–43)	immunoassay	6
		Euthyroid with HF	30	16/14	57.33 ± 3.61	1.64 ± 0.95	3.9 ± 1.3 #	14.8 ± 2.7 #	46 (41–47)		
Kuchulakanti 2019 (26)	India	SHypo with HF	65	27/38	60.8-9.1	>5.50	N/A	normal !	<45	ECLIA	6
		OHypo with HF	42	15/27	61.5-11.8	>5.50	N/A	<0.89 !			
		Euthyroid with HF	243	86/157	60.1-10.2	0.35-5.50	N/A	0.89-1.76 !			
Iacoviello 2020 (27)	Italy	OHypo with HF	190	63/127	67 ± 12	7.79 ± 10.43	2.71 ± 0.57 !	1.63 ± 2.62 !	34 ± 11	N/A	6
		OHyper with HF	59	12/47	63 ± 12	1.05 ± 1.16	3.38 ± 0.96 !	1.32 ± 0.34 !	32 ± 10		
		Euthyroid with HF	498	403/95	62 ± 14	1.63 ± 0.87	3.06 ± 0.41 !	1.79 ± 2.62 !	33 ± 9		
Samuel 2021 (6)	UK	OHypo with HF	312	127/185	74 ± 10	6.40 (5.40-8.50)	N/A	13.0 (11.0-15.0) #	N/A	N/A	7
		OHyper with HF	189	109/80	74 ± 10	0.15 (0.09-0.27)		18.0 (16.0-21.0) #			
		Euthyroid with HF	4491	1161/3330	73 ± 11	1.70 (1.20-2.50)		N/A			
Terlizzese 2021 (28)	Italy	OHypo with HF	83	24/59	64.4 ± 13.47	6.7 ± 9.76	2.6 ± 0.45 !	1.29 ± 0.55 !	32.65 ± 8.77	immunoassay	6
		Euthyroid with HF	174	92/82	61 ± 13	1.67 ± 0.78	2.92 ± 0.36 !	1.22 ± 0.55 !	33.6 ± 8.7		

TD, thyroid dysfunction; SHypo, subclinical hypothyroidism; OHypo, overt hypothyroidism; SHyper, subclinical hyperthyroidism; OHyper, overt hyperthyroidism; HF, heart failure; F, female; M, male; ELICA, electrochemiluminescence immunoassay; ELISA, enzyme-linked immunosorbent assay; LVEF, left ventricular ejection fraction; N/A, unclear. The units of FT3 and FT4 are represented by the symbols *: nom/L, #: pmol/L,!: pg/ml, &:ng/mL.

**Table 2:** The characteristics of the included self-controlled trials that discussed NT-proBNP levels at pre-to-post treatment in TD.

Study, year	Primary disease	N (pre-t/post-t)	Sex(F/M)	Age (range or mean ± sd)	Intervention	TSH(mIU/L) pre-t	TSH(mIU/L) post-t	FT3(pre-t)	FT3(post-t)	FT4(pre-t)	FT4(post-t)	NT-proBNP method
Schultz 2004 (11)	SHypo	21/21	21/0	59 (44–75)	L-T4	16.8 ± 7.24	2.14 ± 1.70	1.43 ± 0.23 $	1.48 ± 0.20$	69.4 ± 12.4 $	107 ± 19.4 $	ECLIA
	OHypo	17/17	17/0	56 (24–76)	L-T4	54 ± 28	3.34 ± 2.24	0.97 ± 0.44 $	1.55 ± 0.45 $	31.3 ± 13.5 $	109 ± 29.1 $	
	SHyper	6/6	6/0	64 (47–81)	RAIT	0.028 ± 0.032	0.6 ± 0.294	2.08 ± 0.18 $	1.81 ± 0.31 $	111 ± 21.2 $	86.3 ± 15.5 $	
	OHyper	13/13	12/1	44(19-71)	ATD and/or RAIT	0.015 ± 0.02	1.58 ± 1.32	5.69 ± 2.18 $	1.73 ± 0.50 $	266 ± 71.1 $	93·4 ± 31.1 $	
Christ 2005 (7)	SHypo	31/31	31/0	58 ± 10	L-T4	11.4 ± 6.6	3.1 ± 1.7	1.9 ± 0.5 *	1.7 ± 0.1 *	11.3 ± 1.9 #	17.4 ± 4.2 #	ECLIA
Bodlaj 2007 (29)	OHyper	59/59	49/10	40.6 ± 14.6	carbimazole	<0.003	2.1 ± 2.4	11.2 ± 4.9!	15.40 ± 0.57!	36.2 ± 17.8!	11.0 ± 2.9!	ECLIA
Hadzovic 2011 (10)	OHypo	35/35	35/0	49.1 ± 4.3	L-T4	13.29 ± 1.09	3.38 ± 0.27	3.34 ± 0.29 *	4.78 ± 0.13*	8.28 ± 0.69 *	15.4 ± 0.57*	ECLIA
	OHyper	34/34	34/0	41.6 ± 10.0	PTU, RAIT	0.03 ± 0.08	0.51 ± 0.12	12.75 ± 1.66*	4.96 ± 0.28*	28.16 ± 2.78*	3.38 ± 0.27*	
Pakuła 2011 (9)	OHypo	24/24	N/A	57.46 ± 15.24	L-T4 100ug/d,39d	29.9 ± 21.9	Normal	1.89 ± 0.89!	Normal!	0.625 ± 0.381!	Normal!	ECLIA
	OHyper	47/47	N/A	52.79 ± 13.74	MMI,30mg/d,14d	0.047 ± 0.122	Normal	11.49 ± 7.01!	Normal!	4.434 ± 2.107!	Normal!	
Scherer 2014 (30)	OHypo	10/10	6/4	46 ± 5	L-T4	85.5 ± 20.3	1.1 ± 0.5	0.6 ± 0.08!	3.7 ± 0.2!	0.09 ± 0.01!	1.8 ± 0.11!	N/A
Muthukumar 2016 (19)	OHyper	41/41	N/A	39.4 ± 8.6	carbimazole or PTU	N/A	N/A	10.2 ± 3.8!	Normal!	2.2 ± 0.9&	Normal&	N/A

TD, thyroid dysfunction; SHypo, subclinical hypothyroidism; OHypo, overt hypothyroidism; SHyper, subclinical hyperthyroidism; OHyper, overt hyperthyroidism; F, female; M, male; L-T4, levothyroxine; PTU, propylthiouracil; MMI, methimazole; RAIT, radioiodine therapy; ATD, anti-thyroid drug; ELICA, electrochemiluminescence immunoassay; ELISA, enzyme-linked immune sorbent assay; N/A, not clear. The units of FT3 and FT4 are represented by the symbols *: nom/L; #: pmol/L; !: pg/ml; &:ng/mL;$:arbitrary units/l.

### Results

#### Overt hyperthyroidism

**Figure 2:**
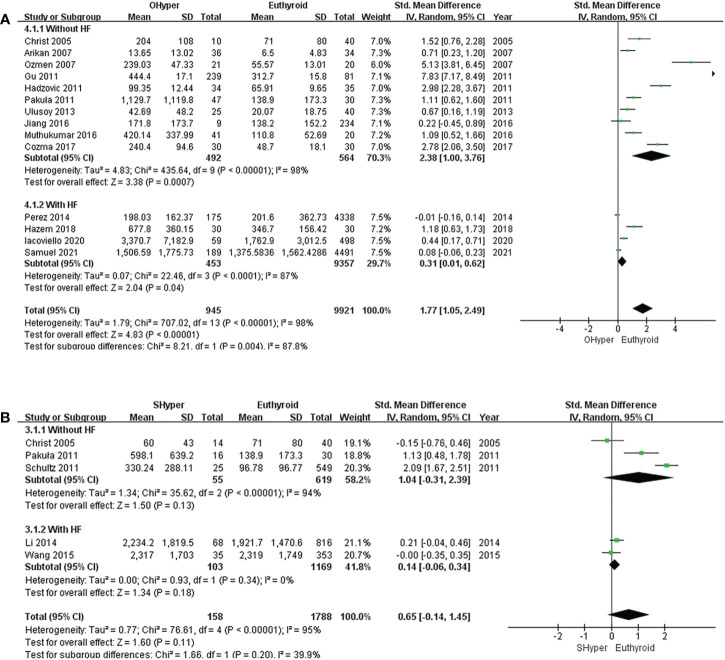
Forest plot of the NT-proBNP levels in patients with **(A)** OHyper and euthyroid subjects; **(B)** SHyper and euthyroid subjects.

The pooled results of the 14 studies showed that NT-proBNP levels were significantly higher in subjects with OHyper than in euthyroid subjects (*I^2^* = 98%, P <0.00001, REM; SMD [95%CI] = 1.77[1.05, 2.49], P <0.00001) (**Figure 2A**). Furthermore, the subgroup analysis indicated that NT-proBNP levels were significantly higher in subjects with OHyper than in euthyroid subjects, regardless of whether they had HF (**Figure 2A**).

#### Subclinical hyperthyroidism

In five studies that compared NT-proBNP levels in SHyper and euthyroid subjects, no significant difference was observed in NT-proBNP levels between SHyper and euthyroid subjects. (*I^2^* = 95%, P <0.00001, REM; SMD [95%CI] = 0.65[-0.14, 1.45], P=0.11; **Figure 2B**). Then, subgroup analyses revealed that patients with SHyper did not significantly differ in NT-proBNP levels, compared with euthyroid subjects, whether participants were suffering from HF or not (**Figure 2B**).

#### Overt hypothyroidism

**Figure 3:**
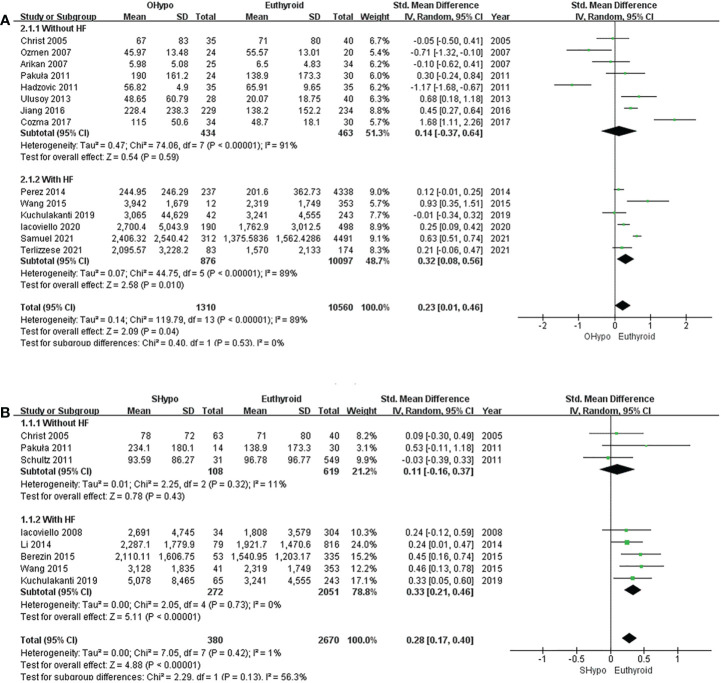
Forest plot of the NT-proBNP levels in patients with **(A)** OHypo and euthyroid subjects; **(B)** SHypo and euthyroid subjects.

The pooled estimate for the 14 studies showed that levels of NT-proBNP were significantly elevated in subjects with OHypo compared to euthyroid subjects (*I^2^* = 89%, P <0.00001, REM; SMD [95%CI] = 0.23 [0.01, 0.46], P=0.04; **Figure 3A**). Subgroup analyses were conducted according to whether the participants had HF, but heterogeneity did not change significantly. No significant difference in NT-proBNP levels was found between patients with OHypo and those with euthyroidism. (*I^2^* = 91%, P <0.00001, REM; SMD [95%CI] = 0.14 [-0.37, 0.64], P=0.59; **Figure 3A**). However, HF patients with OHypo had significantly higher NT-proBNP levels than those with euthyroidism (*I^2^* = 89%, P <0.00001, REM; SMD [95%CI] = 0.32 [0.08, 0.56], P=0.01; **Figure 3A**).

#### Subclinical hypothyroidism

Pooled data from eight studies showed significantly higher NT-proBNP levels among subjects with SHypo than those with euthyroidism. (*I^2^* = 1%, P=0.42, REM; SMD [95%CI] =0.28 [0.17, 0.40], P<0.00001; **Figure 3B**). We conducted a subgroup analysis according to whether participants had HF. NT-proBNP levels in SHypo and euthyroid subjects were not significantly different. (*I^2^* = 11%, P=0.32, REM; SMD [95%CI] = 0.11 [-0.16, 0.37], P=0.43; **Figure 3B**). However, patients with HF and SHypo had significantly higher NT-proBNP levels than those with euthyroid HF. (*I^2^ =*0%, P =0.73, REM; SMD [95%CI] = 0.33 [0.21, 0.46], P<0.00001; **Figure 3B**).

#### Impact of treatment on NT-proBNP levels

**Figure 4:**
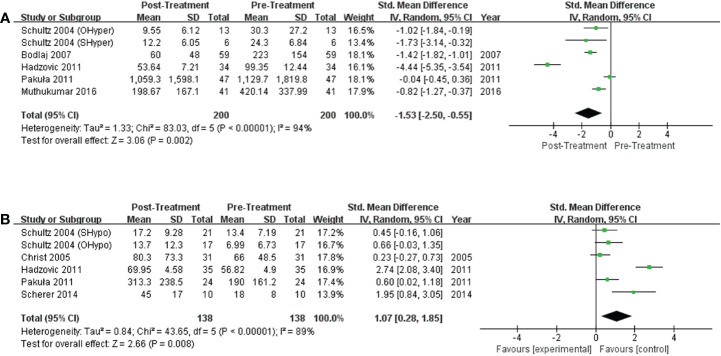
Forest plot of the NT-proBNP levels in subjects with **(A)** hyperthyroidism from pre- to post-treatment; **(B)** hypothyroidism from pre- to post-treatment.

Six studies discussed the effects of treatment on NT-proBNP levels in subjects with hyperthyroidism, and significant decreases in NT-proBNP levels were observed in hyperthyroid patients upon achievement of a euthyroid state. (*I^2^ =*94%, P<0.00001, REM; SMD [95%CI] = -1.53 [-2.50, -0.55], P=0.002; **Figure 4A**). Six trials compared NT-proBNP levels in patients before and after levothyroxine administration. Levothyroxine therapy was related to significantly higher NT-proBNP levels in subjects with hypothyroidism (*I^2^ =*89%, P<0.00001, REM; SMD [95%CI] = 1.07 [0.28, 1.85], P=0.008; **Figure 4B**).

#### Sensitivity analysis and publication bias

The sensitivity analysis showed that the pooled data were similar before and after removing any studies, indicating that the results were relatively stable. Funnel plots were used to determine publication bias. We drew funnel plots for two outcomes (comparing NT-proBNP levels in subjects with OHyper or OHypo and euthyroid controls) that involved more than 10 studies. The funnel plots for OHyper and OHypo are shown in **Figures S1** and **S2**. **Figure S1** is basically symmetric, indicating no significant publication bias. In contrast, **Figure S2** is significantly asymmetrical due to several studies. After we excluded these studies that resulted in the asymmetry of the funnel plot, the result obtained is consistent with the original result, showing that publication bias may not affect our result.

## Discussion

### Overt hyperthyroidism

NT-proBNP levels may increase in patients with OHyper regardless of HF status. The increased NT-proBNP levels may be explained by the following reasons. First, thyroid hormone can directly affect cardiomyocytes and then increase the levels of NT-proBNP. Kohno et al. (31) found that triiodothyronine (T3) and thyroxine (T4) stimulated BNP release in cultured rat ventricular myocytes in a dose-dependent manner. Subsequently, an *in vitro* study by Liang et al. identified the BNP gene as a transcriptional target of thyroid hormone (32). T3 may bind to thyroid hormone receptors (TRs) in the cardiomyocyte nucleus to form a hormone-receptor complex, which regulates BNP transcription by binding to thyroid hormone response elements (TREs) located in approximately 1 kb upstream from the BNP promoter (33–35). In addition, thyroid hormone may activate β-adrenergic signaling (36, 37), and β-adrenergic activation can stimulate BNP mRNA (38–40). In patients with OHyper, specific cardiac-adrenergic receptors are upregulated, and the β-adrenergic responsiveness of cardiomyocytes is increased (36, 37). Anees et al. (2016) found that isoprenaline significantly upregulated BNP (39). In the study by Tshori et al. (2006), β agonists induced protein kinase A (PKA) activity, and PKA has been demonstrated to activate cAMP response element binding protein to increase the expression of microphthalmia transcription factor (MITF), thereby enhancing the activity of BNP promoter (40). Third, OHyper patients are generally in a hypermetabolic state (41–44) and often have various hemodynamic changes, including increased cardiac output, increased heart rate, accelerated blood flow, and increased circulating blood volume, which impacts ventricular pressure (1, 45–48). Secondary changes in blood dynamics can also lead to increased NT-proBNP levels (35, 49). Fourth, OHyper may cause ventricular myocyte structural changes in the heart that conventional echocardiography cannot detect (50), and these alterations may be responsible for NT-proBNP level elevation. In subjects without preexisting cardiac diseases, elevated NT-ProBNP levels in OHyper patients showed only levels comparable to mild HF and may instead signify volume overload than severe HF (8). Moreover, as reported by Hazem et al. (24), increased levels of NT-proBNP in OHyper patients with ischemic heart disease are attributed to the release of BNP stimulated by both OHyper and myocardial ischemic changes, which makes it necessary to check the threshold of NT-proBNP level as a serological marker for the initial diagnosis of HF in this patient group.

### Subclinical hyperthyroidism

According to the meta-analysis, no significant difference was noted between SHyper and euthyroid subjects in NT-proBNP levels, which is inconsistent with the results of some studies. A recent study that investigated the effects of SHyper on BNP levels in 47 patients found that patients with SHyper had higher BNP levels than euthyroid subjects (51). Young adults with serum TSH concentrations below 0.1 mIU/L may experience increased left ventricular (LV) mass, systolic and diastolic LV dysfunction, increased heart rate, and arterial stiffness (52). Thus, the meta-analysis results should be interpreted with caution, given the small number of included studies, relatively small sample sizes, and significant heterogeneity (*I^2^ =*95%, P <0.00001).

### Overt hypothyroidism

The NT-proBNP levels in OHypo and euthyroid subjects without HF did not differ significantly. However, patients with HF and OHypo had significantly elevated NT-proBNP levels compared to HF patients with euthyroid. For the former outcome, Pakula et al. (9) reported that the combined opposite effects of a hypometabolic state brought on by hypothyroidism and increased production of proinflammatory cytokines and endothelins may explain the neutral effect of OHypo on NT-proBNP. Endothelins and proinflammatory cytokines are known to initiate the release of NT-proBNP (53), and there is some proof that thyroid autoimmunity and OHypo cause an inflammatory state and endothelial dysfunction (54–56). However, the underlying cardiac condition may affect the manifestation of TD. In particular, patients with HF may not have sufficient cardiac tolerance to slight changes in thyroid hormone levels. Thus, for patients with HF, small changes, including lowered heart contractility, elevated systemic vascular resistance, impaired left ventricular diastolic filling, and lowered heart output due to OHypo may worsen their preexisting HF, which explains the higher NT-proBNP level in HF patients with OHypo (1, 57–59). Several prospective cohort studies have found that OHypo is an independent risk factor for all-cause mortality and cardiac death among patients with HF (60–62). Therefore, timely and effective treatment of OHypo can improve HF prognosis.

### Subclinical hypothyroidism

According to the meta-analysis, SHypo was not associated with the changes in NT-proBNP levels in patients free from HF. However, Huang et al. (63) found that functional thyroid stimulating hormone receptor (TSHR) was expressed in ventricular tissue and myocytes, and TSH, by acting on TSHR in ventricular myocytes, induced ventricular HMGCR expression *via* the cAMP/PKA/pCREB signaling pathway and promoted BNP secretion to a certain degree. Two studies showed that the plasma BNP level was significantly and positively correlated with the TSH level (18, 64). In addition, some studies showed an inverse association between TSH levels and BNP levels (7, 11, 15, 16). Given the controversy over this point, further studies with larger samples are required. In addition, our meta-analysis demonstrated that HF patients with SHypo had significantly higher levels of NT-proBNP than HF patients with euthyroidism. SHypo may be associated with systolic and diastolic dysfunction, blood pressure alterations, endothelial and vascular dysfunction, and dyslipidemia, which contribute to the development of HF, as reflected by higher NT-proBNP levels (58, 65, 66). According to a recent study, SHypo with TSH ≥7 mIU/L and isolated low T3 levels were related to a poor prognosis over a median of 4.2 years of follow-up in 1365 patients with preexisting HF (67). Randomized controlled trials (RCTs) with placebo controls should be conducted to ascertain the clinical outcomes of treating HF patients with SHypo.

### Treatment of thyroid dysfunction

Interestingly, this meta-analysis showed that the use of levothyroxine increased NT-proBNP levels. This finding is in line with the a forementioned explanation of higher NT-proBNP levels in hyperthyroidism, which is probably associated with the direct effect of exogenous thyroid hormone on the heart. It is still unclear whether levothyroxine therapy is a predisposing factor for HF or whether it aggravates previous HF (65, 66, 68), so more research is needed in the future. In addition, for HF patients receiving levothyroxine therapy, clinicians should closely follow up and pay attention to the occurrence of cardiovascular adverse events in medical practice (69, 70). Moreover, this meta-analysis showed that antithyroid drugs restored the hyperthyroidism-induced increase in plasma NT-pro-BNP level. Therefore, aggressive treatment should be used to avoid severe cardiac complications of hyperthyroidism (atrial fibrillation, heart failure, and embolic events) and reduce the risk of cardiovascular death (57, 71, 72).

### Strengths and limitations

This is the first systematic review to investigate the effects of thyroid disease on NT-proBNP levels. This study has some limitations. First, the observational nature of all the included studies may have affected the validity of the overall results. Second, this systematic review has language bias due to the limited language ability of our researchers, who were unable to access the literature published in languages other than English. Third, given the small number of studies on each prior cardiovascular disease and the recruitment of HF patients with multiple etiologies in some studies, subgroup analyses could not be performed according to the etiology of HF. Patients with HF in all these studies received conventional medical therapy, and there were no significant differences in therapeutic medications. Moreover, the included studies did not further group patients according to the treatment regimens. Thus, we could not perform subgroup analyses according to whether HF patients received treatment or not. Fourth, although this meta-analysis included 24 studies, the sample size was small, considering the wide range of TD. Additionally, more studies with small heterogeneity are needed in the future, considering the significant heterogeneity of most of our outcomes, which may be because several studies did not adjust for important confounders, such as age, sex, and body mass index. In particular, there is controversy about whether gender has a significant effect on BNP (73–78). Only three of the included studies recruited single-sex participants. Based on such a small amount of evidence, we could not further assess whether gender affects BNP, and it therefore needs to be further explored in future studies.

## Conclusions

In conclusion, TD can significantly affect NT-pro-BNP levels, which may change upon reaching a euthyroid state. Notably, the effect of TD on cardiac function may depend on the underlying cardiac status. Thus, timely recognition and effective treatment of cardiac symptoms in patients with TD are mandatory because the prognosis of HF may be improved with appropriate treatment of TD. In the future, RCTs are necessary to examine the prognosis and potential improvement in HF with appropriate treatment of TD.

## Data availability statement

The original contributions presented in the study are included in the article/**Supplementary Material**. Further inquiries can be directed to the corresponding author.

## Author contributions

HZ and LT contributed to the study conception and design. Material preparation, data collection and analysis were performed by HZ, XL, and NZ. The first draft of the manuscript was written by HZ and LT, and all authors commented on previous versions of the manuscript. All authors read and approved the final manuscript.
