# Supplementary material for: Effect of thyroid dysfunction on N-terminal pro-B-type natriuretic peptide levels: A systematic review and meta-analysis
Source: Front Endocrinol (Lausanne). 2023 Jan 26;14:1083171. doi: 10.3389/fendo.2023.1083171 (PMC9909547; doi:10.3389/fendo.2023.1083171)
Supplement: Supplementary file 1 [file DataSheet_1.docx]

**Table** **S1 |** PubMed Search History.

| **No.** | **Query** |
| --- | --- |
| #1 | "thyroid insufficiency"[Title/Abstract] OR "thyroid gland failure"[Title/Abstract] OR "thyroid deficiency"[Title/Abstract] OR "hypothyrosis"[Title/Abstract] OR "hypothyroidosis"[Title/Abstract] OR "Hypothyroidism*"[Title/Abstract] OR "hypothyreosis;"[Title/Abstract] OR "hypothyreoidism*"[Title/Abstract] OR "hyperthyreoidism"[Title/Abstract] OR "hyperthyreosis"[Title/Abstract] OR "hyperthyroidea"[Title/Abstract] OR "Hyperthyroidism*"[Title/Abstract] OR "hyperthyroidosis"[Title/Abstract] OR "thyroid gland hyperfunction"[Title/Abstract] OR "thyroid hyperfunction"[Title/Abstract] OR "thyroideal hyperfunction"[Title/Abstract] OR "thyroidal dysfunction"[Title/Abstract] OR "thyroidal disorder"[Title/Abstract] OR "thyroidal disease"[Title/Abstract] OR "thyroidal anomaly"[Title/Abstract] OR "thyroidal abnormality"[Title/Abstract] OR "thyroid gland dysfunction"[Title/Abstract] OR "thyroid gland disease"[Title/Abstract] OR "thyroid dysfunction*"[Title/Abstract] OR "thyroid disorder*"[Title/Abstract] OR "Thyroid Disease*"[Title/Abstract] OR "thyroid anomaly"[Title/Abstract] OR "thyroid anomalies"[Title/Abstract] OR "thyroid abnormality"[Title/Abstract] OR "thyroid abnormalities"[Title/Abstract] |
| #2 | "Hypothyroidism"[Mesh] |
| #3 | "Hyperthyroidism"[Mesh]" |
| #4 | "Thyroid Diseases"[Mesh] |
| #5 | "Natriuretic Peptide, Brain"[Mesh] |
| #6 | “pro-brain natriuretic peptide" [Supplementary Concept] |
| #7 | "b type natriuretic peptide" OR "BNP 32" OR "BNP Gene Product" OR "Brain Natriuretic Peptide" OR "Brain Natriuretic Peptide 32" OR "Brain Natriuretic Peptide-32" OR "B-Type Natriuretic Peptide" OR "Natrecor" OR "Natriuretic Factor 32" OR "Natriuretic Factor-32" OR "natriuretic peptide B type" OR "Natriuretic Peptide Type B" OR "Natriuretic Peptide Type-B" OR "Nesiritide" OR "Type B Natriuretic Peptide" OR "Type-B Natriuretic Peptide" OR "Ventricular Natriuretic Peptide, B type" OR "Ventricular Natriuretic Peptide, B-type" OR "N-terminal pro-BNP" OR "NT-proBNP" OR "NTproBNP" OR "proBNP (1-76)" OR "N-BNP peptide" OR "NT-BNP" OR "Amino-terminal pro-brain natriuretic peptide" OR "aminoterminal pro-B-type natriuretic peptide" OR "BNP" |
| #8 | (#1 OR #2 OR #3 OR #4) AND (#5 OR #6 OR #7) |

**Table S2 |** Newcastle-Ottawa quality assessment scale (NOS) for the eligible observational cohort, or case-control studies.

| **Study** | **Selection** | | | | **Comparability** | **Exposure** | | | **Score** |
| --- | --- | --- | --- | --- | --- | --- | --- | --- | --- |
|  | Is the case definition adequate? | Representativeness of the cases | Selection of Controls | Definition of Controls | Control for most important factor | Ascertainment of exposure | Same method of ascertainment for cases and controls | Non-Response rate |  |
| Christ 2005 | ***** | ***** | ***** | ***** | ***** | ***** | ***** | ***** | 8 |
| Arikan 2007 | ***** |  | ***** | ***** | ***** | ***** | ***** | ***** | 7 |
| Ozmen 2007 | ***** |  | ***** |  | ***** | ***** | ***** | ***** | 6 |
| Gu 2011 | ***** | ***** |  | ***** | ****** | ***** | ***** | ***** | 8 |
| Hadzovic 2011 | ***** |  |  | ***** | ***** | ***** | ***** | ***** | 6 |
| Pakuła 2011 | ***** |  |  | ***** |  | ***** | ***** | ***** | 5 |
| Schultz 2011 | ***** | ***** |  | ***** |  | ***** | ***** | ***** | 6 |
| Ulusoy 2013 | ***** |  | ***** | ***** |  | ***** | ***** | ***** | 6 |
| Jiang 2016 | ***** | ***** |  | ***** |  | ***** | ***** | ***** | 6 |
| Muthukumar 2016 | ***** | ***** |  | ***** | ***** | ***** | ***** | ***** | 7 |
| Cozma 2017 | ***** | ***** | ***** | ***** | ***** | ***** | ***** | ***** | 8 |
| Iacoviello 2008 | ***** | ***** |  | ***** |  | ***** | ***** | ***** | 6 |
| Li 2014 | ***** | ***** |  | ***** |  | ***** | ***** | ***** | 6 |
| Perez 2014 | ***** | ***** |  | ***** | ***** | ***** | ***** | ***** | 7 |
| Berezin 2015 | ***** | ***** |  | ***** | ***** | ***** | ***** | ***** | 7 |
| Wang 2015 | ***** | ***** |  | ***** |  | ***** | ***** | ***** | 6 |
| Hazem 2018 | ***** |  |  | ***** |  | ***** | ***** | ***** | 6 |
| Kuchulakanti 2019 | ***** |  |  | ***** | ***** | ***** | ***** | ***** | 6 |
| Iacoviello 2020 | ***** | ***** |  | ***** |  | ***** | ***** | ***** | 6 |
| Samuel 2021 | ***** | ***** |  | ***** | ***** | ***** | ***** | ***** | 7 |
| Terlizzese 2021 | ***** | ***** |  | ***** |  | ***** | ***** | ***** | 6 |

**Table S3 |** The Prior cardiovascular disease and cardiovascular therapy in patients with heart failure.

| Study, year | Etiology of heart failure or the prior cardiovascular disease | Treatment of heart failure |
| --- | --- | --- |
| Iacoviello 2008 | Patients with chronic heart failure of any origin who had been receiving conventional medical therapy for at least three months and were in stable condition. The exclusion criteria were acute heart failure, a coronary artery by-pass graft or myocardial infarction within the previous three months. | conventional medical therapy. |
| Li 2014 | Patients were admitted due to decompensation symptoms, and physical signs of heart failure and dilated cardiomyopathy were defined as systolic dysfunction (LVEF<50%) with Left ventricle dilation. secondary cardiomyopathies were excluded from the study: ischemic heart disease, alcohol-induced cardiomyopathy, congenital heart disease, and rheumatic heart disease. | conventional medical therapy: diuretics, ACEI/ARB, beta-blockers, digoxin, aspirin, anticoagulant. |
| Perez 2014 | Patients who were at least 60 years of age with symptomatic (NYHA II to IV), ischemic, systolic (LVEF]＜40%) heart failure. | conventional medical therapy: diuretics, ACEI or ARB, beta-blocker, digitalis glycoside, antiarrhythmic therapy, anticoagulant therapy. |
| Berezin 2015 | Patients with documented ischemia-induced chronic heart failure who underwent angiography or PCI, as well as post-myocardial infarction subjects with LVEF of less than 45%. | conventional medical therapy: ACEI or ARB, aspirin, beta-adrenergic blockers, dihydropyridine calcium channel blockers, ivabradine, mineralocorticoid receptor antagonists, diuretics. |
| Wang 2015 | Dilated cardiomyopathy (DCM), DCM was based on the echocardiographic findings of a large left ventricle with end-diastolic diameter greater than 56 mm and LV dysfunction with ejection fraction less than 45%. | conventional medical therapy: ACEI/ARB, beta-blocker, aldosterone antagonists,diuretics. |
| Hazem 2018 | Ischemic heart disease. The exclusion criteria: congenital heart diseases, pericarditis, myocarditis, acute myocardial infarction or acute cerebral infarction,advanced diseases of the liver or kidneys (glomerular filtration rate <30 ml/min/1.73 m2), and the presence of artificial pacemaker, inflammatory or infectious diseases, and malignant neoplasms. | conventional medical therapy. |
| Kuchulakanti 2019 | Hospitalized patients presenting with acute heart failure for the first time.The exclusion criteria: recurrent heart failure, severe kidney injury (creatinine plasma level above 4.9 mg/dL), pulmonary oedema, valvular heart disease, thyrotoxicosis, tachyarrhythmia, ischaemic stroke. | conventional medical therapy. |
| Iacoviello 2020 | Prior cardiovascular disease: ischemic cardiomyopathy, atrial fibrillation hypertension. | conventional medical therapy: ACEI/ARBs, beta-blockers, diuretics, aldosterone antagonists, amiodarone. |
| Samuel 2021 | Prior cardiovascular disease: ischemic heart disease, hypertension, chronic obstructive pulmonary disease. | conventional medical therapy: diuretic, ACEI or ARB, beta-blockers, mineralocorticoid receptor antagonist, statin. |
| Terlizzese 2021 | Prior cardiovascular disease:Ischemic cardiomyopathy, atrial fibrillation, hypertension. | conventional medical therapy: ACEI or ARB, beta-blockers, amiodarone, diuretics. |

**Abbreviations:** LVEF, left ventricular ejection fraction; ACEI, angiotensin converting enzyme inhibitor; ARB, angiotensin receptor blocker.

**Table S4 |** The JBI critical appraisal checklist for quasi-experimental studies (the self-controlled trials).

|  | Schultz 2004 | Christ 2005 | Bodlaj 2007 | Hadzovic 2011 | Pakuła 2011 | Scherer 2014 | Muthukumar 2016 |
| --- | --- | --- | --- | --- | --- | --- | --- |
| Is it clear in the study what is the cause and what is the effect？ | Yes | Yes | Yes | Yes | Yes | Yes | Yes |
| Were the participants included in any comparisons similar? | Yes | Yes | Yes | Yes | Yes | Yes | Yes |
| Were the participants included in any comparisons receiving similar treatment/care, other than the exposure or intervention of interest? | Yes | Yes | Yes | Yes | Yes | Yes | Yes |
| Was there a control group? | NO | NO | NO | NO | NO | NO | NO |
| Were there multiple measurements of the outcome both pre and post the intervention/exposure? | NO | Yes | NO | NO | Yes | Yes | Yes |
| Was follow up complete and if not, were differences between groups in terms of their follow up adequately described and analyzed? | Yes | Yes | unclear | unclear | unclear | Yes | Yes |
| Were the outcomes of participants included in any comparisons  measured in the same way? | Yes | Yes | Yes | Yes | Yes | Yes | Yes |
| Were outcomes measured in a reliable way? | Yes | Yes | Yes | Yes | Yes | Yes | Yes |
| Was appropriate statistical analysis used? | Yes | Yes | Yes | Yes | Yes | Yes | Yes |


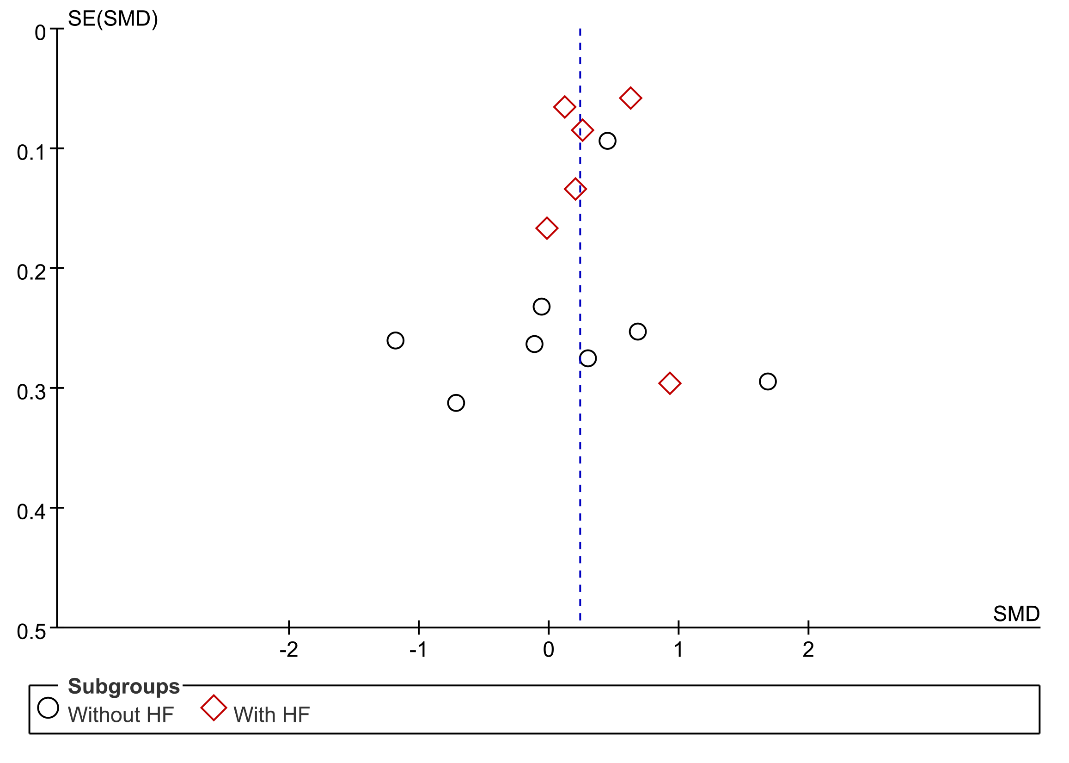


**Figure S1 |** Funnel plot of studies comparing NT-proBNP levels between OHypo subjects and euthyroid controls.


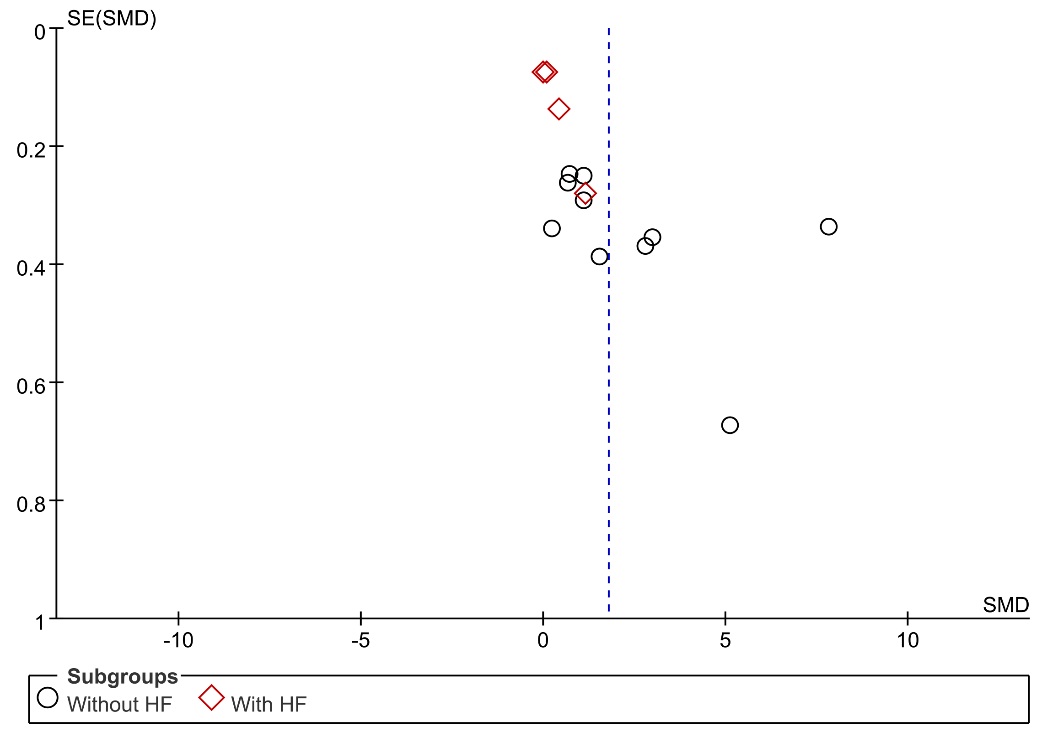


**Figure S2 |** Funnel plot of studies comparing NT-proBNP levels between OHyper subjects and euthyroid controls.
